# Examination of Diurnal Variation and Sex Differences in Hippocampal Neurophysiology and Spatial Memory

**DOI:** 10.1523/ENEURO.0124-22.2022

**Published:** 2022-11-08

**Authors:** Lacy K. Goode, Allison R. Fusilier, Natalie Remiszewski, Jacob M. Reeves, Kavitha Abiraman, Matthew Defenderfer, Jodi R. Paul, Lori L. McMahon, Karen L. Gamble

**Affiliations:** 1 Department of Psychiatry and Behavioral Neurobiology, University of Alabama at Birmingham Heersink School of Medicine, Birmingham 35233, AL; 2 Research Computing, Information Technology, University of Alabama at Birmingham, Birmingham 35233, AL; 3 Department of Cell, Developmental, and Integrative Biology, University of Alabama at Birmingham, Birmingham 35233, AL; 4 Prescott Medical Communications Group, Chicago 60601, IL

**Keywords:** circadian, hippocampus, memory, plasticity, rhythms, synaptic

## Abstract

Circadian rhythms are biological processes that cycle across 24 h and regulate many facets of neurophysiology, including learning and memory. Circadian variation in spatial memory task performance is well documented; however, the effect of sex across circadian time (CT) remains unclear. Additionally, little is known regarding the impact of time-of-day on hippocampal neuronal physiology. Here, we investigated the influence of both sex and time-of-day on hippocampal neurophysiology and memory in mice. Performance on the object location memory (OLM) task depended on both circadian time and sex, with memory enhanced at night in males but during the day in females. Long-term synaptic potentiation (LTP) magnitude at CA3-CA1 synapses was greater at night compared with day in both sexes. Next, we measured spontaneous synaptic excitation and inhibition onto CA1 pyramidal neurons. Frequency and amplitude of inhibition was greater during the day compared with night, regardless of sex. Frequency and amplitude of excitation was larger in females, compared with males, independent of time-of-day, although both time-of-day and sex influenced presynaptic release probability. At night, CA1 pyramidal neurons showed enhanced excitability (action potential firing and/or baseline potential) that was dependent on synaptic excitation and inhibition, regardless of sex. This study emphasizes the importance of sex and time-of-day in hippocampal physiology, especially given that many neurologic disorders impacting the hippocampus are linked to circadian disruption and present differently in men and women. Knowledge about how sex and circadian rhythms affect hippocampal physiology can improve the translational relevancy of therapeutics and inform the appropriate timing of existing treatments.

## Significance Statement

Circadian rhythms regulate many aspects of neurophysiology, including cognition. However, the impact of time-of-day and sex on hippocampal neurophysiology and hippocampus-dependent memory remains largely unexplored. Here, we report that circadian regulation of object location memory (OLM) is sex dependent. Furthermore, examination of hippocampal physiology across time-of-day in both sexes revealed: enhanced long-term synaptic potentiation at night, greater daytime inhibitory synaptic transmission onto CA1 pyramidal neurons, effects of both sex and time-of-day on excitatory synaptic transmission onto CA1 pyramidal neurons, and enhanced nighttime excitability of CA1 pyramidal neurons that is dependent on both synaptic input and position along anterior-posterior hippocampal axis. These results underscore the importance of accounting for sex, regional location, and time-of-day in the study of hippocampal physiology.

## Introduction

The hippocampus is the seat of learning and memory in the brain and its primary output is generated by the principal cells (i.e., pyramidal neurons) in area CA1. Action potential firing by a CA1 pyramidal neuron, like any other neuron, is a combined function of excitatory and inhibitory synaptic drive, intrinsic membrane properties regulating excitability, and neuromodulators (Spruston, 2008). A relatively unexplored facet in the hippocampus is how CA1 pyramidal neuron physiology is modulated by time-of-day. At the cellular level, time-of-day variations in biological function are generated by a transcriptional-translational feedback loop (Partch et al., 2014). Tissue-clocks throughout the body are hierarchically organized in a system that drives the timing of 24-h rhythms in physiology and behavior, enabling organisms to adapt to and anticipate regularly occurring events in their environment (Pilorz et al., 2020; Buijs et al., 2021). Circadian regulation of physiological processes is advantageous, and dysregulation of circadian rhythms can promote and exacerbate disease onset and symptoms (Logan and McClung, 2019; Colwell, 2021). Therefore, understanding circadian influence on physiology is crucial for designing interventions for diseases with circadian dysfunction, such as neurodegenerative diseases (Lee et al., 2021). Moreover, the majority of foundational knowledge concerning fundamental principles of hippocampal physiology is based on studies conducted in nocturnal, mostly male, rodents during their inactive phase (daytime). While the scientific community has begun to address the importance of sex as a factor in biomedical research, the importance of time-of-day is still relatively underemphasized. The overarching goal of this study was to begin to unveil how sex and time-of-day interact to influence daily variation in hippocampal physiology and function.

The suprachiasmatic nucleus (SCN) of the hypothalamus is the principal orchestrator of the endogenous circadian network, and electrical properties of SCN neurons vary across time-of-day. In fact, circadian regulation of neuronal excitability is widespread in the mammalian brain (Paul et al., 2020) and has been observed in a range of species, including rodents (Snider et al., 2018), *Drosophila* (Cao and Nitabach, 2008; Sheeba, 2008), and zebrafish (Elbaz et al., 2013). Although the SCN is the principal clock, autonomous circadian clocks exist in other brain regions, including hippocampus (Paul et al., 2020; Hartsock and Spencer, 2020). At the molecular level, subregions of hippocampus rhythmically express core clock proteins, with the cell body layer of area CA1 having the strongest expression of PER2 (Jilg et al., 2010). Moreover, over 600 genes, including those encoding ion channels and synaptic proteins exhibit circadian expression in hippocampus (Zhang et al., 2014; Renaud et al., 2015). At the cellular level, long-term potentiation (LTP), a form of plasticity in which specific patterns of synaptic stimulation results in a long-lasting increase in the strength of synaptic transmission, is expressed at a greater magnitude at night compared with day in nocturnal mice (Chaudhury et al., 2005; Besing et al., 2017; Davis et al., 2020). Cognitive function is also regulated by the circadian system (Wright et al., 2012) and circadian regulation of performance on hippocampus-dependent memory assays has been demonstrated across several species (Snider et al., 2018). However, our understanding of how sex affects circadian regulation of cognition is limited. Furthermore, evidence at the cellular level is lacking, including a detailed understanding of how time-of-day and sex regulate synaptic drive onto and membrane properties of CA1 pyramidal cells. Here, we sought to determine how sex and time-of-day modulate the hippocampal circuit: from the behavioral level down to individual neuronal physiology. We found that circadian regulation of hippocampus-dependent memory is dependent on sex, while day-night differences in hippocampal LTP are not. We also found that synaptic transmission and neuronal excitability vary as a function of time-of-day and uncovered that some of these changes depend on sex.

## Materials and Methods

### Animals

All animal procedures followed the *Guide for the Care and Use of Laboratory Animals*, United States Public Health Service and were approved by the University of Alabama at Birmingham Institutional Animal Care and Use Committee. All experiments used 6- to 12-week-old C57BL/6J mice of both sexes obtained from Jackson Laboratories (http://jaxmice.jax.org/strain/013636.html) or from the C57BL/6J colony at University of Alabama at Birmingham. Mice were maintained on a 12/12 h light/dark cycle with *ad libitum* access to food (LabDiet Rodent 5001 by Purina) and water. Mice were group housed in same-sex cages of four mice for behavior experiments. For all other experiments, mice were group housed in same-sex cages of two to seven mice per cage.

### Object location memory (OLM)

The object location memory (OLM) task (Snider et al., 2016) was conducted under <10 lux dim red light. Four cohorts of mice were used, with each cohort consisting of eight males and eight females. Within each cohort, mice were assigned to undergo habituation, training, and testing either during the day or during the night. In cohorts 1 and 3, males were tested during the day, and females were tested at night. In cohorts 2 and 4, females were tested during the day and males were tested at night.

**Figure 1.**
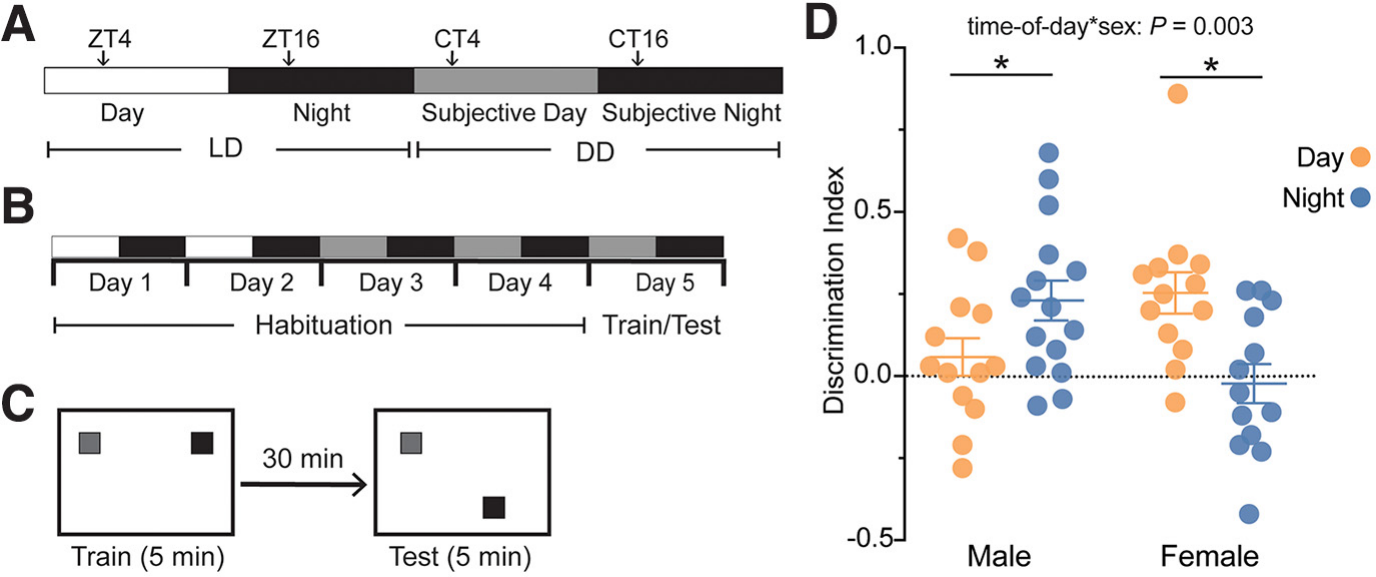
Circadian rhythms regulate object location memory (OLM) performance in a sex-dependent manner. ***A***, Schematic illustrating light-dark (LD) cycle and constant dark (DD) cycle terminology. ZT is used to denote time points in LD where ZT 12 refers to lights off. CT is used to denotate time points in DD where CT 12 is the projected night from the prior LD cycle. Experiments were conducted at ZT 4 or CT 4 (day) and ZT 16 or CT 16 (night). ***B***, Experimental timeline illustrating habitation during LD and DD on days 1–4 and training and testing procedures in DD on day 5. ***C***, Schematic of OLM experimental procedure. Mice were placed in arena and allowed to explore two objects during 5-min training phase at either CT 4 or CT 16. After a 30-min delay in the home cage, one object remained in the same location in the arena (familiar), and one object was moved to a new location (novel), and mice were placed back in the arena and allowed to explore for the 5-min testing phase. ***D***, Scatterplot displaying all individual discrimination index scores with mean ± SEM. Data were plotted for two time points and both sexes: male night, *n = *13; male day, *n *=* *13; female day, *n *=* *15; female night, *n *=* *13 (*interaction, *p *=* *0.023, two-way ANOVA; *time-of-day for males, *p *=* *0.047, simple main effects; *time-of-day for females, *p *=* *0.003, simple main effects). In all plots, blue codes for observations at night, and orange codes observations made during the day. ZT, Zeitgeber time; CT, Circadian time. SEM, standard error of the mean. n, number of mice. Total exploration time was not different across groups and did not predict DI scores (Extended Data Fig. 1-1). For a detailed statistical summary, see Extended Data Table 1-1.

Mice were entrained to a 12/12 h light/dark (LD) cycle, and then habituated to the arena for 2 d at either Zeitgeber time (ZT) four or ZT 16 (where ZT 12 refers to lights off), for day and night, respectively (days 1–2; Fig. 1*A*,*B*). After day 2, mice were released into constant darkness (DD) and again habituated to the arena at projected circadian time (CT) 4 or 16 (days 3–4, where CT 12 refers to the projected time of lights off from the prior LD cycle; Fig. 1*A*,*B*). Mice were allowed to acclimate to the behavior room for 20 min each day immediately before habituation or train/test. Habituation consisted of 5 min of handling followed by 5 min of arena exploration with visual cues present. Visual cues consisted of vertical stripes on one wall and a large red X on another wall. The arenas were 35.5 × 25.4 cm with 20.3-cm-high walls. OLM training and testing occurred in DD, 24 h after the final day of habituation at projected CT four or CT 16. Objects were made with PRETEX Building Blocks (Item No. 8030-100) and had three possible positions within the arena, all at least 8.9 cm away from the walls.

10.1523/ENEURO.0124-22.2022.f1-1 Extended Data Figure 1-1 Total exploration is not different across groups and does not predict OLM performance. ***A***, Total exploration time for individual mice during the testing phase with mean ± SEM across day and night in both sexes (time-of-day: ns *p *=* *0.926, sex: ns *p *=* *0.936, interaction: ns *p *=* *0.692, two-way ANOVA). There was no difference in distribution of high explorers (total exploration time > 35 s) and low explorers (total exploration time < 35 s) across four groups (*p *=* *0.619, Pearson’s χ^2^). ***B***, Correlation between total exploration times and DI scores during test phase (ns *p *=* *0.704, *r* = –0.053, Pearson’s correlation). Male night (dark blue) *n = *13 mice; male day (light blue), *n *=* *13 mice; female day (light green), *n *=* *15 mice; female night (dark green), *n = *13 mice. For a detailed statistical summary, see Extended Data Table 1-1. Download Figure 1-1, TIF file

During training, each mouse was allowed to explore an arena with two objects for 5 min. Afterwards, the mouse was returned to its home cage for 30 min, during which one of the objects from the original exploration was moved to a new position (the novel location) while one remained in its original position (the familiar location; Fig. 1*C*). A 30-min recall period was chosen based on previously published methods (Snider et al., 2016) and to avoid memory interference because of sleep deprivation or memory enhancement from an overnight sleep period. In the test phase, each mouse was placed back in the arena with the novel and familiar location objects and allowed to explore for 5 min. All habituation, training, and testing were recorded at 30 FPS (ELP Camera Model: ELP-USBFHD05MT-KL36IR). Exploration was tracked using a computer model made via DeepLabCut. Object interaction was then analyzed using custom MATLAB (MathWorks) scripts developed by Mary Phillips (https://github.com/PhillipsML/DLC-NovelObject#dlc-novelobject). For data analysis, several exclusion criteria were applied: mice that exhibited a clear side preference, mice that spent most of their time exploring objects for the purpose of trying to escape the arena, and mice with a high preference to one object over the other during training were excluded. Discrimination index was calculated as: (time spent exploring novel object location − time spent exploring familiar object location)/(time spent exploring novel object location + time spent exploring familiar object location).

### Electrophysiology

#### Slice preparation

Mice were killed with cervical dislocation and rapid decapitation at ZT 0–1 or ZT 11–12 for day and night experiments, respectively. Both sex and time-of-day were interleaved. For extracellular field experiments, brains were removed and 350-μm coronal slices were prepared using a VT1200 S vibratome (Leica Biosystems) in an ice-cold solution containing the following (in mm): 85 NaCl, 2.5 KCl, 4 MgSO_4_ * 7 H_2_O, 0.5 CaCl_2_ * 2H_2_O, 1.25 NaH_2_PO_4_, 75 Sucrose, 25 NaHCO_3_, 25 Glucose saturated in 95% O_2_ and 5% CO_2_. Slices were allowed to rest for at least 1 h in a recovery solution of standard artificial CSF (ACSF) containing the following (in mm): 119 NaCl, 2.5 KCl, 1.3 MgSO_4_ * 7H_2_O, 2.5 CaCl_2_ * 2H_2_O, 1 NaH_2_PO_4_, 26 NaHCO_3_, and 11 glucose, bubbled with 95% O_2_/5% CO_2_. For whole-cell patch-clamp experiments, brains were removed and 300-μm thick coronal slices were prepared using a VT1200 S vibratome (Leica Biosystems) in an ice-cold solution containing the following (in mm): 110 choline chloride, 25 glucose, 7 MgCl_2_, 2.5 KCl, 1.25 Na_2_PO_4_, 0.5 CaCl_2_, 1.3 Na-ascorbate, 3 Na-pyruvate, and 25 NaHCO_3_, bubbled with 95% O_2_/5% CO_2_. Slices were allowed to rest for at least 1 h at room temperature in a recovery solution containing the following (in mm): 125 NaCl, 2.5 KCl, 1.25 Na2PO_4_, 2 CaCl_2_, 1 MgCl_2_, 25 NaHCO_3_, and 25 glucose, bubbled with 95% O_2_/5% CO_2_. For experiments measuring inhibitory synaptic events, 2 mm kynurenic acid was added to the recovery solution.

#### Field recordings

Data were obtained from ZT 1–6 or ZT 12–18 for day and night recordings, respectively. Coronal hippocampal slices were placed in a submersion chamber and continuously perfused with standard ACSF at 3–5 ml/min and 26–28°C. Schaffer collateral axons were stimulated using a bipolar stimulating electrode placed in stratum radiatum of area CA3. Field EPSPs (fEPSPs) were obtained with a recording electrode placed in stratum radiatum of area CA1, within 200–300 μm of the stimulating electrode. The initial slope of the fEPSPs (fEPSP slope) was measured at the linear region immediately following the fiber volley and preceding the fEPSP peak. Data were acquired and analyzed using pCLAMP10/11(Molecular Devices). Data were recorded using a Kerr Scientific S2 amplifier (Kerr Tissue Recording System, Kerr Scientific Instruments). Signals were digitized at 10 kHz (Digidata 1550B).

Input-output (I/O) curves were generated by measuring the slope of fEPSPs from CA1 stratum radiatum in response to a series of increasing stimulation intensities (0.2–200 μA, Δ 10 μA) at the Schaffer Collaterals. Baseline fEPSPs were obtained by delivering a 0.1-Hz stimulation to elicit fEPSPs of approximately −0.20 mV/ms for 20 min.

Long-term potentiation (LTP) experiments were conducted by obtaining and maintaining a stable baseline fEPSP response for 20 min, and then LTP was induced by delivering a high-frequency stimulation (HFS; 100 Hz;0.5 s duration; delivered 2× with 15-s interval). This weaker stimulation protocol was chosen to avoid masking a day/night difference in LTP magnitude (Besing et al., 2017; Davis et al., 2020). After HFS, fEPSP slopes were recorded for 40 min.

#### Whole-cell patch-clamp recordings

All data were collected from coronal hippocampal slices during ZT 2–6 (day) or ZT 13–17 (night) at 32°C in standard ACSF containing the following (in mm): 125 NaCl, 2.5 KCl, 1.25 Na_2_PO_4_, 2 CaCl_2_, 1 MgCl_2_, 25 NaHCO_3_, and 25 glucose, bubbled with 95% O_2_/5% CO_2_. Whole-cell patch-clamp recordings of CA1 pyramidal neurons were obtained using the blind patch technique. Briefly, patch pipettes were placed at either the medial or lateral end of area CA1 (dependent on whether a slice was from the left or right hemisphere) at a depth of ∼50–150 μm, positive pressure was applied as the pipette was slowly advanced either medially or laterally through the pyramidal cell layer until a rapid increase in pipette resistance indicated contact with a neuron, at which point positive pressure was released, a tight seal (>1 GΩ) was obtained, and slight negative pressure was applied to achieve whole-cell patch configuration. Data were acquired using a Multiclamp 700B amplifier, Axon Digidata 1440A and 1550B digitizer, and pClamp10/11 software (Molecular Devices). Patch pipettes (BF150–086; Sutter Instruments) were pulled on a Sutter P-97 horizontal puller (Sutter Instruments) to a resistance between 2.5 and 5 MΩ. Cells were dialyzed for 5 min before experimental recordings. Cells used for analysis had access resistance <30 MΩ that did not increase by >20% for the duration of each 5-min experiment.

For voltage-clamp experiments, all cells were held at −70 mV, and signals were filtered at 5 kHz and digitized at 10 kHz. IPSCs experiments used a patch pipette solution containing (in mm): 140 CsCl, 10 EGTA, 5 MgCl_2_, 2 Na-ATP, 0.3 Na-GTP, 10 HEPES, 0.2% biocytin (pH 7.3, 290 mOsm), and 5 QX-314 (sodium channel antagonist) added at time of use. IPSCs were pharmacologically isolated with bath perfusion of 10 μm NBQX (AMPAR antagonist, Hello Bio) and 5 μm CPP (NDMAR antagonist, Hello Bio). EPSCs experiments used a patch pipette solution containing (in mm): 100 CsOH, 100 gluconic acid (50%), 0.6 EGTA, 5 MgCl_2_, 2 Na-ATP*3H_2_O, 0.3 Na-GTP, 40 HEPES, 7 phosphocreatine, biocytin (0.2%), and 5 QX-314 added at time of use. EPSCs were pharmacologically isolated with bath perfusion of 10 μm gabazine (GABA_A_R antagonist, Hello Bio). Separate experiments to measure miniature IPSC and miniature EPSCs (mIPSCs/mEPSCs) were recorded as above with the addition 0.5 μm tetrodotoxin (TTX; voltage-gated sodium channel inhibitor, Tocris).

For current clamp experiments, signals were filtered at 10 kHz and digitized at 20 kHz. Patch pipette solution contained (in mm): 135 K-Gluconate, 2 MgCl_2,_ 0.1 EGTA, 10 HEPES, 4 KCl, 2 Mg-ATP, 0.5 Na-GTP, 10 phosphocreatine, and biocytin (0.2%; pH 7.3, 310 mOsm, and 2–4 MΩ). Neuronal excitability was assessed by injecting progressive steps of depolarizing current from rest (0–500 pA, Δ 20 pA) and counting the number of action potentials fired during each 1000 ms current step. The response slope was obtained by calculating the linear relationship between firing frequency and injected current across 160- to 400-pA steps. The maximum action potential (AP) firing frequency (max) and current at which max occurred (Imax) were also measured. Sag was measured as the amplitude (mV) of the peak voltage from a hyperpolarizing current injection that achieved a steady-state membrane potential of 90–93 mV. Input resistance (MΩ) was measured as the slope of the current response to a series of hyperpolarizing current injections (−150 to 0 pA, Δ 50 pA). Rheobase was defined as the minimum current required to elicit a single AP. Single APs elicited by rheobase were used to analyze action potential properties (Tables 1, 2). AP amplitude was defined as the voltage difference between AP threshold and its peak. Threshold was defined as the voltage (mV) at which the AP first derivative (dV/dt) exceeded 20 mV/s. AP rise time was the time (ms) for an AP to reach 90% of its peak amplitude from 10% of its peak. Decay time was the time between 90% and 10% of AP peak amplitude. Half-width was the time (ms) between the half amplitudes of the rise and decay of the AP waveform. Afterhyperpolarization (AHP) was the difference between baseline and the most hyperpolarized point occurring within 3 ms after AP threshold for fast-AHP (fAHP) and 10–50 ms after AP threshold for medium-AHP (mAHP). Peak AP rise and fall was defined as the maximum slope (ΔmV/Δms) for AP rise and decay, respectively. Baseline membrane potential was calculated as the mean voltage over the 1400-ms sweep during the 0-pA step. Initial experiments were done in the absence of synaptic blockers to determine how sex and time-of-day contribute to CA1 pyramidal neuron excitability in the intact circuit. To begin to assess the influence of synaptic transmission on enhanced nighttime excitability, a separate, follow-up experiment was conducted in the presence of the GABA_A_ antagonist, gabazine (10 μm), and the glutamatergic antagonists, NBQX (10 μm) and CPP (5 μm).

**Table 1:** Membrane properties of CA1 pyramidal neurons across day and night

		Day	Night
		Mean ± SEM (*n*, *N*)	Mean ± SEM (*n*, *N*)
Baseline (mV)	Anterior	−70.57 ± 0.84 (33,11)	−69.79 ± 0.78 (33,14)
	Posterior^a^	−69.99 ± 0.94 (27,11)	−67.53 ± 0.82 (31,11)
Input resistance (MΩ)	Anterior	63.73 ± 2.84 (33,11)	64.81 ± 2.71 (33,14)
	Posterior	73.3 ± 3.43 (27,11)	78.16 ± 3.53 (31,11)
Sag amplitude (mV)	Anterior	4.84 ± 0.36 (33,11)	4.94 ± 0.33 (33,14)
	Posterior	5.63 ± 0.47 (27,11)	6.43 ± 0.41 (31,11)
AP response slope	Anterior	0.053 ± 0.004 (33,11)	0.051 ± 0.005 (33,14)
	Posterior	0.052 ± 0.005 (27,11)	0.046 ± 0.01 (31,11)
Max (Hz)	Anterior	16.58 ± 0.96 (33,11)	18.27 ± 0.84 (33,14)
	Posterior	18.37 ± 1.06 (27,11)	19.42 ± 0.818 (31,11)
Imax (pA)	Anterior	418.18 ± 13.96 (33,11)	416.36 ± 12.45 (33,14)
	Posterior	394.07 ± 15.54 (27,11)	380.65 ± 15.120 (31,11)
Single action potential properties			
Anterior	Rheobase (pA)	153.28 ± 10.67 (29,11)	139.35 ± 10.51 (26,11)
	Amplitude (mV)	91.82 ± 0.80 (29,11)	93.52 ± 0.76 (26,11)
	Threshold (mV)	−43.37 ± 0.66 (29,11)	−43.45 ± 0.59 (26,11)
	Rise time (ms)^b^	0.29 ± 0.01 (29,11)	0.28 ± 0.01 (26,11)
		Male: 0.30 ± 0.01 (28,11)	
		Female: 0.28 ± 0.01 (27,11)	
	Decay time (ms)	0.93 ± 0.02 (29,11)	0.92 ± 0.02 (26,11)
	Half-width (ms)	0.93 ± 0.02 (29,11)	0.94 ± 0.01 (26,11)
	fAHP (mV)	−6.42 ± 0.49 (29,11)	−6.24 ± 0.48 (26,11)
	mAHP (mV)	−9.68 ± 0.51 (29,11)	−8.98 ± 0.49 (26,11)
	Peak rise (ΔmV/Δms)	400.25 ± 11.60 (29,11)	414.86 ± 10.03 (26,11)
	Peak fall (ΔmV/Δms)	−98.34 ± 1.98 (29,11)	−99.48 ± 1.62 (26,11)
Posterior	Rheobase (pA)	120.56 ± 8.75 (27,11)	120.56 ± 8.75 (27,11)
	Amplitude (mV)	91.45 ± 0.81 (27,11)	91.45 ± 0.81 (27,11)
	Threshold (mV)	−42.58 ± 0.59 (27,11)	−42.58 ± 0.59 (27,11)
	Rise time (ms)	0.32 ± 0.01 (27,11)	0.32 ± 0.01 (27,11)
	Decay time (ms)	1.14 ± 0.03 (27,11)	1.14 ± 0.03 (27,11)
	Half-width (ms)	1.11 ± 0.03 (27,11)	1.11 ± 0.03 (27,11)
	fAHP (mV)	−5.01 ± 0.43 (27,11)	−5.01 ± 0.43 (27,11)
	mAHP (mV)	−8.25 ± 0.41 (27,11)	−8.25 ± 0.41 (27,11)
	Peak rise (ΔmV/Δms)	352.36 ± 10.63 (27,11)	352.36 ± 10.63 (27,11)
	Peak fall (ΔmV/Δms)	−80.31 ± 2.70 (27,11)	−80.31 ± 2.70 (27,11)

a significant main effect of time-of-day, *p *=* *0.044, two-way ANOVA.

b significant main effect of sex, *p *=* *0.047, two-way ANOVA.

See Extended Data Table 1-1 for detailed statistical summary. n = number of cells, N = number of mice.

10.1523/ENEURO.0124-22.2022.t1-1 Extended Data Table 1-1 Statistical summary. Download Table 1-1, XLSX file

**Table 2:** Membrane properties of CA1 pyramidal neurons across day and night in synaptic antagonists

		Day	Night
		Mean ± SEM (*n*, *N*)	Mean ± SEM (*n*, *N*)
Baseline (mV)	Anterior	−68.41 ± 1.55 (10,6)	−67.42 ± 1.37 (11,6)
	Posterior	−68.14 ± 1.33 (14,6)	−67.44 ± 1.38 (13,6)
Input resistance (MΩ)	Anterior	74.38 ± 5.14 (10,6)	74.49 ± 8.76 (11,6)
	Posterior	74.26 ± 4.76 (14,6)	74.28 ± 5.88 (13,6)
Sag amplitude (mV)	Anterior	6.37 ± 0.58 (10,6)	6.37 ± 0.65 (11,6)
	Posterior	6.43 ± 0.54 (14,6)	6.36 ± 0.48 (13,6)
AP response slope	Anterior	0.048 ± 0.01 (10,6)	0.04 ± 0.015 (11,6)
	Posterior	0.051 ± 0.009 (14,6)	0.059 ± 0.011 (13,6)
Max (Hz)	Anterior	18.30 ± 1.46 (10,6)	19.55 ± 0.71 (11,6)
	Posterior	20.07 ± 1.05 (14,6)	21.62 ± 1.06 (13,6)
Imax (pA)	Anterior	346.0 ± 29.22 (10,6)	370.91 ± 14.11 (11,6)
	Posterior	381.43 ± 20.91 (14,6)	407.69 ± 19.68 (13,6)
Single action potential properties			
Anterior	Rheobase (pA)	132.7 ± 14.76 (10,6)	121.82 ± 12.82 (11,6)
	Amplitude (mV)	93.17 ± 1.15 (10,6)	92.885 ± 1.44 (11,6)
	Threshold (mV)	−40.28 ± 0.90 (10,6)	−42.15 ± 0.65 (11,6)
	Rise time (ms)	0.29 ± 0.01 (10,6)	0.32 ± 0.02 (11,6)
	Decay time (ms)	1.02 ± 0.04 (10,6)	1.01 ± 0.03 (11,6)
	Half-width (ms)	0.99 ± 0.02 (10,6)	0.98 ± 0.01 (11,6)
	fAHP (mV) ^a^^,^^b^	−5.77 ± 0.74 (10,6)	−4.08 ± 0.51 (11,6)
		Male: −5.83 ± 0.56 (11,6)	
		Female: −3.98 ± 0.58 (10,6)	
	mAHP (mV)^c^	−9.29 ± 0.65 (10,6)	−7.06 ± 0.59 (11,6)
	Peak rise (ΔmV/Δms)	406.80 ± 20.07 (10,6)	397.56 ± 18.27 (11,6)
	Peak fall (ΔmV/Δms)	−92.16 ± 3.01 (10,6)	−91.97 ± 1.78 (11,6)
Posterior	Rheobase (pA)	126.07 ± 11.17 (14,6)	115.23 ± 14.59 (13,6)
	Amplitude (mV)	92.90 ± 1.03 (14,6)	92.07 ± 1.46 (13,6)
	Threshold (mV)	−40.82 ± 0.49 (14,6)	−41.01 ± 0.55 (13,6)
	Rise time (ms)	0.29 ± 0.01 (14,6)	0.28 ± 0.01 (13,6)
	Decay time (ms)	1.08 ± 0.03 (14,6)	1.04 ± 0.02 (13,6)
	Half-width (ms)	1.03 ± 0.02 (14,6)	1.00 ± 0.02 (13,6)
	fAHP (mV)	−5.13 ± 0.58 (14,6)	−5.72 ± 0.45 (13,6)
	mAHP (mV)	−8.67 ± 0.66 (14,6)	−9.65 ± 0.54 (13,6)
	Peak rise (ΔmV/Δms)	390.30 ± 12.53 (14,6)	406.47 ± 16.78 (13,6)
	Peak fall (ΔmV/Δms)	−86.65 ± 2.31 (14,6)	−88.15 ± 1.96 (13,6)

a significant main effect of time-of-day, *p *=* *0.044, two-way ANOVA.

b significant main effect of sex, *p *=* *0.034, two-way ANOVA.

c significant main time-of-day, *p *=* *0.024, two-way ANOVA. n = number of cells, N = number of mice.

### Immunohistochemistry

#### Biocytin

To confirm that cells recorded to measure postsynaptic currents were CA1 pyramidal cells, all cells were filled with biocytin for at least 20 min. Slices containing filled cells were fixed in 4% paraformaldehyde for at least 24 h, then washed for 3 × 10 min in PBS, and incubated for 2–3 h at RT in a TBS solution containing 10% NDS, 3% BSA, 1% glycine, 0.4% Triton X-100, and streptavidin-488 (1:1000). Slices were then washed for 3 × 10 min in PBS and mounted on glass slides and coverslips with ProLong Gold Antifade mounting media containing DAPI. Slides were visualized on a BZ-X700 fluorescence microscope (Keyence). Any cells that could not be classified as CA1 pyramidal cells based on location and morphology were excluded from analysis.

### Analysis and statistics

Data were analyzed and visualized using SPSS (version 27/28) and Prism-GraphPad software. Assumptions of parametric tests, including normality and homogeneity of variance were assessed, and if violated, data were transformed, or nonparametric tests were used. Unless otherwise stated, significance was ascribed at *p *<* *0.05. A summary of all statistical tests is provided in Extended Data Table 1-1.

#### Object location memory

OLM data were analyzed using an independent two-way ANOVA with time-of-day and sex as independent variables and discrimination index as the dependent variable (Fig. 1*D*). A Pearson’s correlation was used to assess the relationship between total exploration time and discrimination index scores and a contingency analysis was used to determine the distribution of high versus low exploration times across sex and time-of-day (Extended Data Fig. 1-1*B*).

#### Field recordings

Input-output data were analyzed using a linear mixed model with fEPSP slope as a function of Time-of-day, Sex, and Stimulation Intensity. For LTP experiments, the fEPSP slopes were normalized to the baseline responses, and responses obtained during the last 10 min of the 40-min post-HFS recording period were analyzed using a three-way ANOVA with repeated measures (RM-ANOVA).

#### Whole-cell patch-clamp electrophysiology

Postsynaptic currents (inhibitory and excitatory) were automatically detected from a 5 min recording using pClamp’s event template search then manually inspected for false event detection. The amplitudes and interevent interval (IEI) were analyzed using a generalized estimating equation (GEE) that allowed parameter estimates with population-averaged models while taking into account correlations between repeated measures within subjects (Reed and Kaas, 2010; Cook et al., 2016). The GEE model specified an unstructured working correlation matrix structure, a subject effect of cell, and a within-subject effect of postsynaptic events. The raw data had a significant positive skew with extreme values and thus, were trimmed of upper and lower outliers (10%) followed by either a log transformation in the case of the amplitude data, or a log + 1 transformation in the case of IEI data, to meet assumptions of normal distributions before analysis.

All current-clamp data were analyzed with Easy Electrophysiology (Easy Electrophysiology, RRID:SCR_021190), a software package that utilizes Neo (Garcia et al., 2014). Action potentials were counted using the Action Potential Counting module with the default, Auto-Threshold Spike algorithm. A RM-ANOVA was used to analyze action potentials across current steps in which data did not violate the assumptions of linearity and normality: 160–400 pA. All other membrane properties (Tables 1, 2) were analyzed using a two-way ANOVA with independent variables of time-of-day and sex. All current-clamp data were stratified by position along the anterior-posterior axis before final statistical analysis.

## Results

### Day-night differences in OLM performance depend on sex

To examine the effect of sex on circadian rhythms of learning and memory, we used the object location memory (OLM) assay, which relies on a mouse’s tendency to explore objects in novel locations, to assess hippocampal spatial memory (Barker and Warburton, 2011; Takahashi et al., 2013; Chao et al., 2016). Although circadian and diurnal differences in performance on OLM have been reported (Takahashi et al., 2013; Snider et al., 2016), the effect of sex on diurnal variation in OLM performance remains poorly understood. We found that OLM performance varies across time-of-day; however, the pattern of diurnal difference in performance differed between sexes (*p = *0.023, two-way ANOVA interaction). While males performed better at night compared with the day, as expected (*p = *0.028, simple main effects comparing day vs night in males; Fig. 1*D*), female mice performed better during the day compared with night (*p = *0.004, simple main effects comparing day vs night in females; Fig. 1*D*). There was no effect of time-of-day or sex on total exploration time (*p = *0.926 and 0.936, two-way ANOVA main effects; Extended Data Fig. 1-1*A*). There was no relationship between total exploration and DI scores (*r*_(52)_ = −0.053, ns *p *=* *0.704, Pearson’s correlation; Extended Data Fig. 1-1*B*).

### LTP magnitude at night is greater than day, regardless of sex

Long-term potentiation (LTP) is considered a cellular correlate of learning and memory. LTP at CA3-CA1 synapses is higher at night compared with the day in male mice (Chaudhury et al., 2005; Besing et al., 2017; Davis et al., 2020), but to our knowledge, there are no published reports of the effects of time-of-day on LTP magnitude in adult female mice. Given our finding that diurnal differences in performance on a hippocampal-dependent memory assay are dependent on sex, we next sought to determine whether sex affects diurnal differences in LTP.

**Figure 2.**
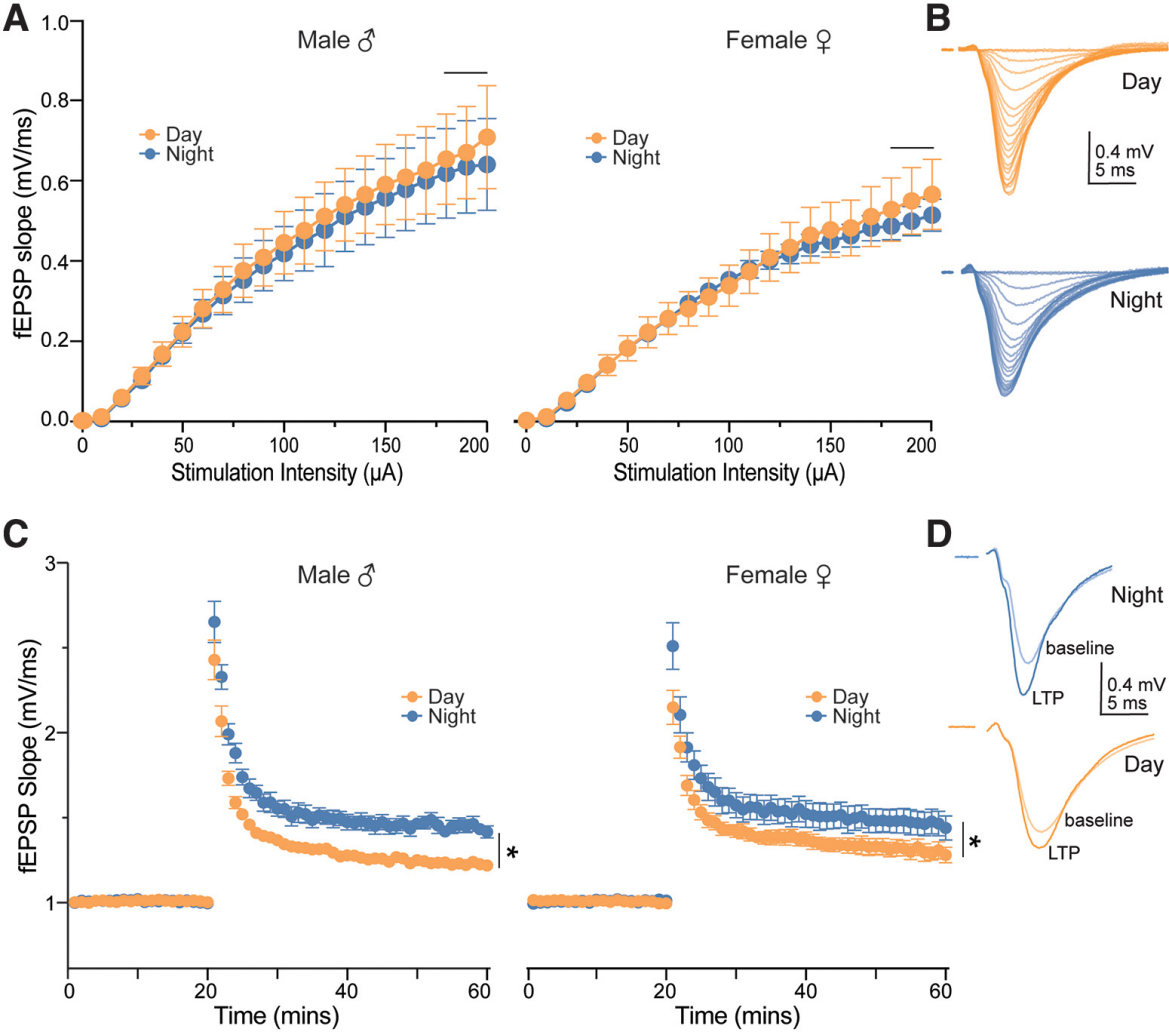
LTP magnitude is greater at night compared with the day, regardless of sex. ***A***, Average slopes of fEPSPs in response to increasing stimulation of the Schaffer collaterals (time-of-day × sex interaction: *p *<* *0.001). Note that significant sex differences in input-output responses were observed only at 180 μA (*p *=* *0.041), 190 μA (*p *=* *0.042), and 200 μA (*p *=* *0.035), regardless of time-of-day, as indicated by horizontal line above stimulation intensities. Data were plotted for two time points and both sexes: male day, *n *=* *13 slices from 3 mice; male night, *n* =10 slices from 3 mice; female day, *n *=* *14 slices from 3 mice; female night, *n *=* *12 slices from 3 mice. ***B***, Example fEPSPs from two male mice used to generate input-output curves in ***A***. ***C***, Average slopes of fEPSPs before and after a HFS (100 Hz, 0.5 s, 2×; *t* = 20 min) to Schaffer collaterals (time-of-day: *p =* *0.003; means ± SEMs at 60 min, night: 1.451 ± 0.176; day: 1.286 ± 0.141). Data were plotted for two time points and both sexes: male day, *n *=* *7 slices from 3 mice; male night, *n *=* *8 slices from 3 mice; female day, *n *=* *10 slices from 3 mice; female night, *n *=* *9 slices from 3 mice. ***D***, Example fEPSPs from two female mice used to produce LTP in C. In all plots, blue codes recordings at night and orange codes recordings during the day. ♀ = female. ♂ = male. LTP, Long-term potentiation; fEPSPs, field EPSPs; SEM, standard error of the mean; HFS, high-frequency tetanus. All statistical tests were performed with a three-way linear mixed model (input-output curves) or three-way, RM-ANOVA (LTP). Data are shown as means ± SEMs. For a detailed statistical summary, see Extended Data Table 1-1.

First, to assess the strength of basal synaptic transmission at CA3-CA1 synapses, we generated I/O curves by measuring the fEPSP slope from CA1 stratum radiatum in response to Schaffer collateral stimulation over a series of increasing stimulation intensities (0.2–200 μA, Δ 10 μA) during the day and night in male and female mice (Fig. 2*A*,*B*). While neither sex nor time-of-day had a significant effect on basal synaptic transmission over the stimulus range tested (*p = *0.552 and 0.981, respectively, LMM main effect), there was a significant sex by stimulation intensity interaction (*p *<* *0.001, LMM; Fig. 2*A*,*B*). Males had larger fEPSP slopes compared with females only at 180,190, and 200 μA, regardless of time-of-day (*p = *0.041, 0.043, and 0.035, respectively; simple main effects comparing males and females across all stim intensities; Fig. 2*A*). Overall, these results indicate that time-of-day does not affect basal synaptic transmission and that sex affects responses only at the highest stimulation intensities.

Next, we assessed synaptic plasticity at the CA3-CA1 synapse by measuring LTP in response to a brief, high-frequency stimulation (HFS; Fig. 2*C*,*D*). As previously reported, the magnitude of LTP was greater at night compared with the day in both male and female mice (*p *= 0.003, three-way RM-ANOVA; Fig. 2*C*,*D*); however, there was no significant effect of or interaction with sex. Together, these findings suggest that time-of-day affects synaptic plasticity in male and female mice, without influencing basal synaptic strength.

### Synaptic inhibition onto CA1 pyramidal cells during the day is greater than night, regardless of sex

**Figure 3.**
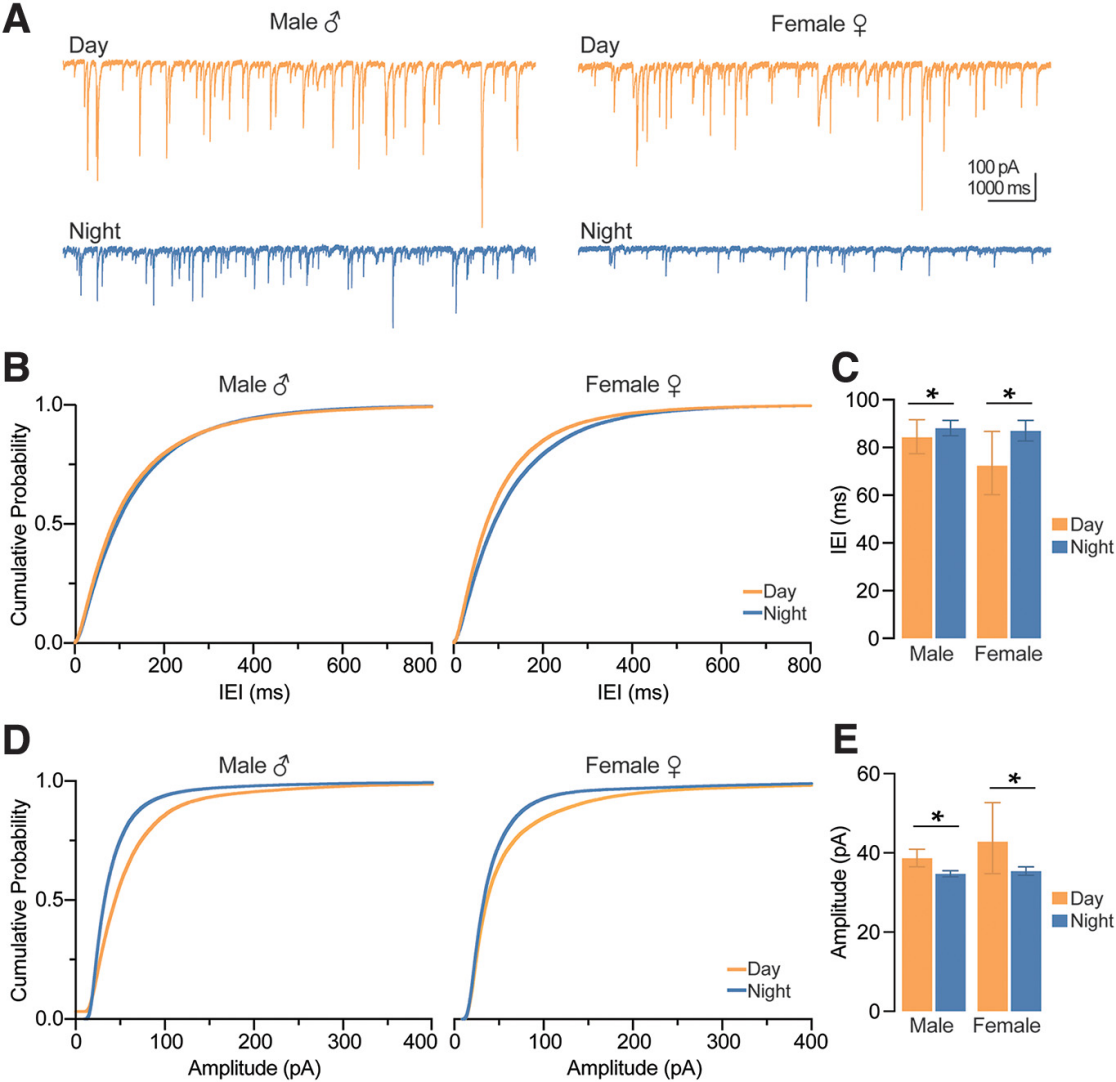
Action potential-mediated inhibition onto CA1 pyramidal neurons is greater during the day compared with night, regardless of sex. ***A***, Example traces of IPSCs onto CA1 pyramidal neurons. Scale bars represent 100 pA and 1000 ms. ***B***, Cumulative probability distribution plots for the IEI of sIPSCs. ***C***, Estimated marginal means and confidence intervals of the IEI of sIPSCs; time-of-day: *p =* *0.003; pooled EMM [95% confidence intervals] for night, 87.491 [82.215, 90.180] ms and day, 78.050 [72.164, 87.145] ms. ***D***, Cumulative probability distribution plots for the amplitude of sIPSCs. ***E***, Estimated marginal means and confidence intervals of the amplitude of sIPSCs; time-of-day: *p =* *0.008; pooled EMM [95% confidence intervals] for day, 41.697 [37.523, 46.334] pA and night, 36.066 [35.424, 36.720] pA. sIPSCs were measured from five male mice during the day (*n *=* *16 cells), five male mice at night (*n *=* *28 cells), five female mice during the day (*n *=* *18 cells), and five female mice at night (*n *=* *17 cells). In all plots, blue codes recordings at night and orange codes recordings during the day. ♀ = female. ♂ = male. EMM, estimated marginal means; IEI, interevent interval; sIPSCs, spontaneous IPSCs. All statistical tests were performed with a two-way GEE model. Data were shown as EMM ± confidence intervals. For a detailed statistical summary, see Extended Data Table 1-1.

Changes in LTP can be attributed to synaptic mechanisms and/or intrinsic changes in excitability. Therefore, we first sought to determine whether time-of-day and sex affect inhibitory and excitatory synaptic transmission onto CA1 pyramidal neurons. To examine synaptic inhibition onto CA1 pyramidal cells, we measured the amplitude and frequency of spontaneous IPSCs (sIPSCs) using whole-cell voltage clamp in male and female mice during the day and night (Fig. 3*A*). We found that sIPSC interevent interval (IEI) during the day was shorter than at night, regardless of sex (time-of-day: *p *=* *0.033, GEE; Fig. 3*A*,*C*), indicating a greater frequency of inhibitory events during the day. The amplitude of sIPSCs during the day was larger than night in both males and females (time-of-day: *p *=* *0.008, GEE; Fig. 3*A*,*E*). This increased day-time frequency and amplitude of sIPSCs suggest stronger inhibition onto CA1 pyramidal neurons during the day compared with night.

**Figure 4.**
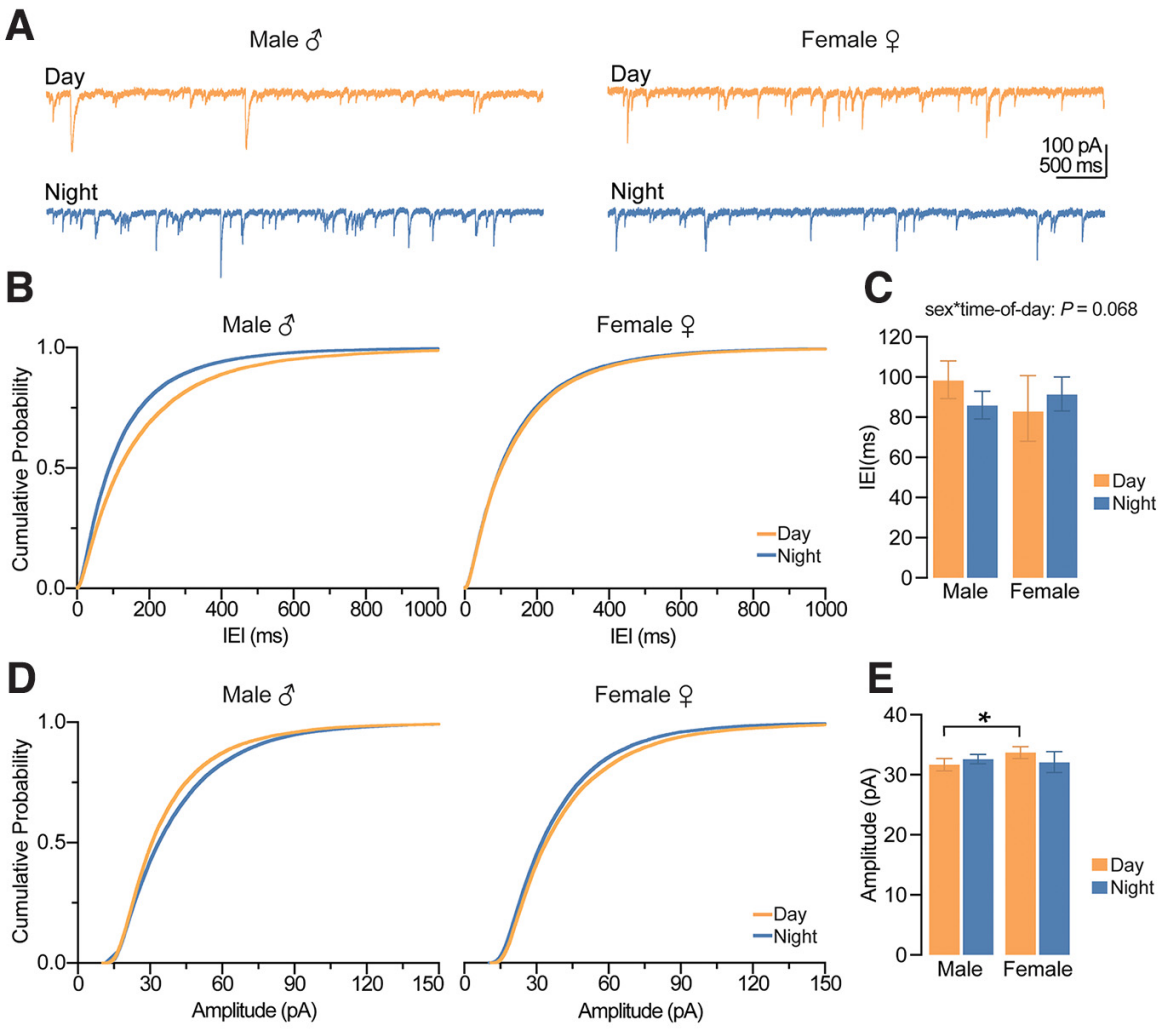
Action potential-independent inhibition onto CA1 pyramidal neurons is influenced by sex and time-of-day. ***A***, Example traces of spontaneous, mIPSCs onto CA1 pyramidal neurons. Scale bars represent 100 pA and 500 ms. ***B***, Cumulative probability distribution plots for the IEI of mIPSCs. ***C***, Estimated marginal means and confidence intervals of the IEI of mIPSCs (interaction, *p *=* *0.068). ***D***, Cumulative probability distribution plots for the amplitude of mIPSCs. ***E***, Estimated marginal means and confidence intervals of the amplitude of mIPSCs (interaction: *p = 0.038). Note that significant sex differences were observed during the day (*p *=* *0.006) but not at night (*p *=* *0.594). mIPSCs were measured from male five mice during the day (*n *=* *12 cells), five male mice at night (*n *=* *13 cells), five female mice during the day (*n *=* *12 cells), and five female mice at night (*n *=* *14 cells). In all plots, blue codes recordings at night and orange codes recordings during the day. ♀ = female. ♂ = male. EMM, estimated marginal means; IEI, interevent interval; mIPSCs, miniature IPSCs. All statistical tests were performed with a two-way GEE model followed by Wald χ^2^ pairwise comparisons. Data were shown as EMM ± confidence intervals. For a detailed statistical summary, see Extended Data Table 1-1.

Stronger synaptic inhibition during the day could arise from an increase in presynaptic GABA release, or from increased postsynaptic GABA_A_R function. To distinguish between these possibilities, we measured miniature IPSCS (mIPSCs) in the presence of the voltage-gated sodium channel blocker tetrodotoxin (TTX) in both male and female mice during the day and night (Fig. 4*A*). While we found that neither sex (*p *=* *0.392, GEE) nor time-of-day (*p *=* *0.760, GEE) had a statistically significant effect on mIPSC IEI, events from males trended toward exhibiting a day-night difference (*p *=* *0.068, sex × time-of-day interaction, GEE; Fig. 3*C*). The lack of day-night differences in females indicates that the time-of-day effects on sIPSCs is likely driven by local interneuron action potential firing. In males, the mean values between day and night differed by ∼12 ms (mean and SEM: male day, 98.24 ± 1.05 ms; male night, 85.78 ± 1.04 ms), suggesting that action potential-independent inhibitory vesicle release may be more frequent at night (Fig. 3*C*). When we examined mIPSC amplitude, we unexpectedly found a significant interaction between time-of-day and sex (*p *=* *0.038, GEE), with amplitudes in females being larger than males only during the day (*p *=* *0.006, Wald χ^2^ pairwise comparisons; Fig. 4*E*); however, this ∼2-pA difference is likely not biologically relevant (female-day: 34.68 ± 1.01 pA; male-day: 32.67 ± 1.02 pA, mean ± SEM).

Taken together, these spontaneous and miniature IPSC data suggest that action potential-dependent inhibition, but not spontaneous vesicle fusion, onto CA1 pyramidal cells is greater during the day compared with night in both males and females.

### Synaptic excitation onto CA1 pyramidal cells depends on sex

**Figure 5.**
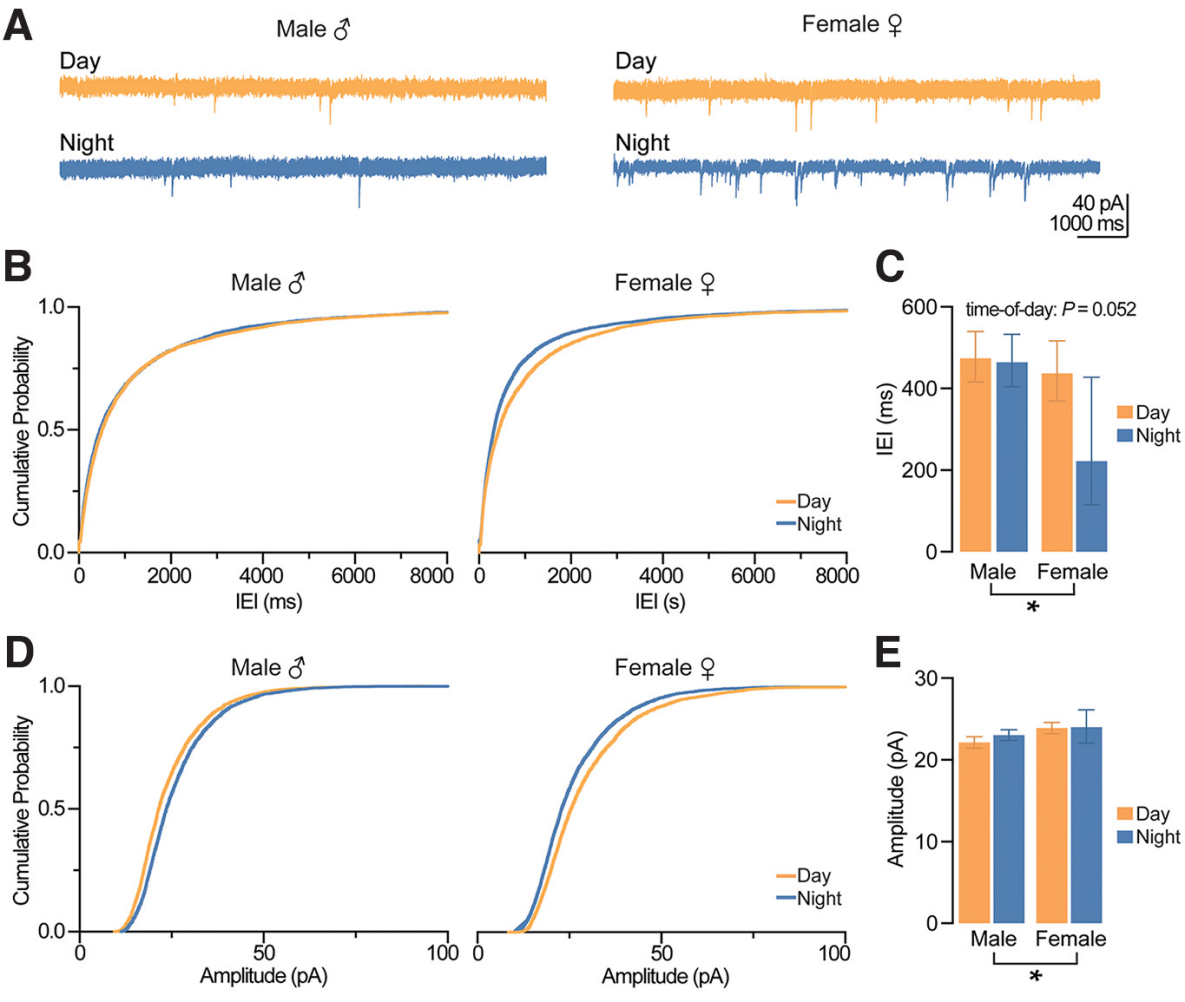
Action potential-mediated excitation onto CA1 pyramidal neurons depends on sex. ***A***, Example traces of spontaneous sEPSCs onto CA1 pyramidal neurons. Scale bars represent 40 pA and 1000 ms. ***B***, Cumulative probability distribution plots for the IEI of sEPSCs. ***C***, Estimated marginal means ± confidence intervals of the IEI of sEPSCs; sex: *p =* *0.022; pooled EMM [95% confidence intervals] for males, 469.002 [426.705, 515.530] ms and females, 311.536 [222.006, 436.968] ms. Note that differences in sEPSC IEIs recorded during the day (455.037 [410.344, 506.913] ms) and night (321.107 [230.600, 449.839] ms) failed to reach statistical significance (time-of-day: *p *=* *0.052). ***D***, Cumulative probability distribution plots for the amplitude of sEPSCs. ***E***, EMM and confidence intervals of the amplitude of sEPSCs; sex: *p =* *0.020; pooled EMM [95% confidence intervals] for females, 24.952 [23.898, 26.046] pA and males, 23.578 [23.111, 24.058] pA. sEPSCs were recorded from five male mice during the day (*n *=* *17 cells), five male mice at night (*n *=* *16 cells), five female mice during the day (*n *=* *16 cells), and five female mice at night (*n *=* *18 cells). In all plots, blue codes recordings at night and orange codes recordings during the day. ♀ = female. ♂ = male. EMM, estimated marginal means; IEI, interevent interval; sEPSCs, spontaneous EPSCs. All statistical tests were performed with a two-way GEE model. Data were shown as EMM ± confidence intervals. For a detailed statistical summary, see Extended Data Table 1-1.

We next wanted to determine whether spontaneous excitatory synaptic input onto CA1 pyramidal neurons was affected by sex and time-of-day. First, we measured spontaneous EPSCs (sEPSCs) using whole-cell voltage-clamp recordings (Fig. 5*A*). Although there was no significant main effect of time-of-day on sEPSC amplitude (Fig. 5*E*), regardless of sex (*p *=* *0.371, GEE), a statistical trend for a significant main effect of time-of-day indicated that sEPSC IEI recorded during the day may be greater than those recorded at night (*p *=* *0.052, GEE), suggesting a greater frequency of excitatory events at night (Fig. 5*C*). Overall, we found that females had more excitatory synaptic input, with larger sEPSC amplitudes and shorter IEIs compared with males (*p *=* *0.022 and 0.020, respectively, main effect of sex, GEE; Fig. 5*C*,*E*).

**Figure 6.**
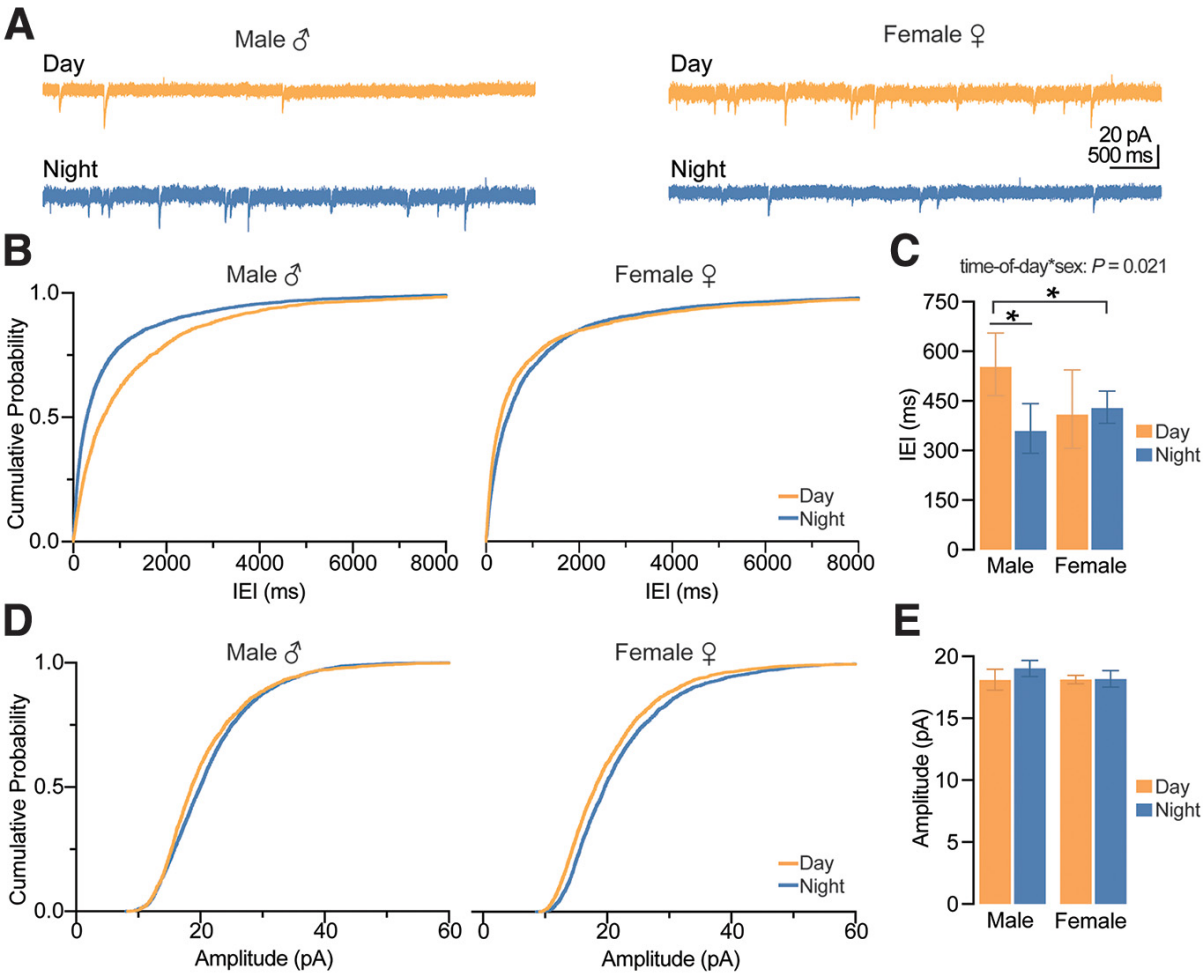
Action potential-independent excitation onto CA1 pyramidal neurons is dependent on sex and time-of-day. ***A***, Example traces of spontaneous mEPSCs onto CA1 pyramidal neurons. Scale bars represent 20 pA and 500 ms. ***B***, Cumulative probability distribution plots for the IEI of mEPSCs. ***C***, Estimated marginal means and confidence intervals of the IEI of mEPSCs (interaction: *p =* *0.021). Note that significant time-of-day differences were observed in males (*p =* *0.002) but not in females (*p *=* *0.765). ***D***, Probability distribution plots for the amplitude of mEPSCs. ***E***, EMM and confidence intervals of the amplitude of mEPSCs (no significant effects). Spontaneous mEPSCs were recorded from four male mice during the day (*n *=* *11 cells), three male mice at night (*n *=* *13 cells), three female mice during the day (*n *=* *14 cells), and four female mice at night (*n *=* *13 cells). In all plots, blue codes recordings at night and orange codes recordings during the day. ♀ = female. ♂ = male. EMM, estimated marginal means; IEI, interevent interval; mEPSCs, miniature EPSCs. All statistical tests were performed with a two-way GEE model followed by Wald χ^2^ pairwise comparisons. Data were shown as EMM ± confidence intervals. For a detailed statistical summary, see Extended Data Table 1-1.

Next, we repeated these experiments in the presence of the voltage-gated sodium channel blocker tetrodotoxin (TTX) and measured miniature spontaneous excitatory synaptic currents (mEPSCs) onto CA1 pyramidal neurons (Fig. 6*A*). Amplitude of mEPSCs did not vary across sex or time-of-day (*p *=* *0.227 and *p *=* *0.150, main effect of sex and time-of-day, respectively, GEE; Fig. 6*E*); however, day-night variation in mEPSC IEIs was dependent on sex (*p *=* *0.021, time-of-day by sex interaction, GEE; Fig. 6*C*). In males, mEPSC IEIs were shorter at night than during the day (*p *=* *0.002, Wald χ^2^ pairwise comparisons), indicating a greater frequency of excitatory events at night in male mice and therefore a likely increase in presynaptic release probability; however, there was no significant day-night difference in females (*p *=* *0.765, Wald χ^2^ pairwise comparisons; Fig. 6*C*).

Taken together, these results suggest that the trend toward increased nighttime sEPSC frequency (especially in females) is action potential dependent. However, in the males, blocking action potentials uncovers a nighttime increase in frequency that was not seen in the sEPSCs.

### CA1 pyramidal neurons are more excitable at night

Broadly, our observations suggest that synaptic inhibition is greater at night and synaptic excitation is greater during the day; thus, we next wanted to determine whether this opposing diurnal variation in synaptic excitatory and inhibitory input results in diurnal variation in CA1 pyramidal neuron excitability. To this end, we patched CA1 pyramidal cells in current clamp mode with the circuit intact (i.e., in the absence of synaptic antagonists) and without clamping cell membrane potential. We injected increasing amounts of depolarizing current (0–500 pA, Δ 20 pA, 1000-ms duration) into pyramidal neurons and measured the number of action potentials elicited.

**Figure 7.**
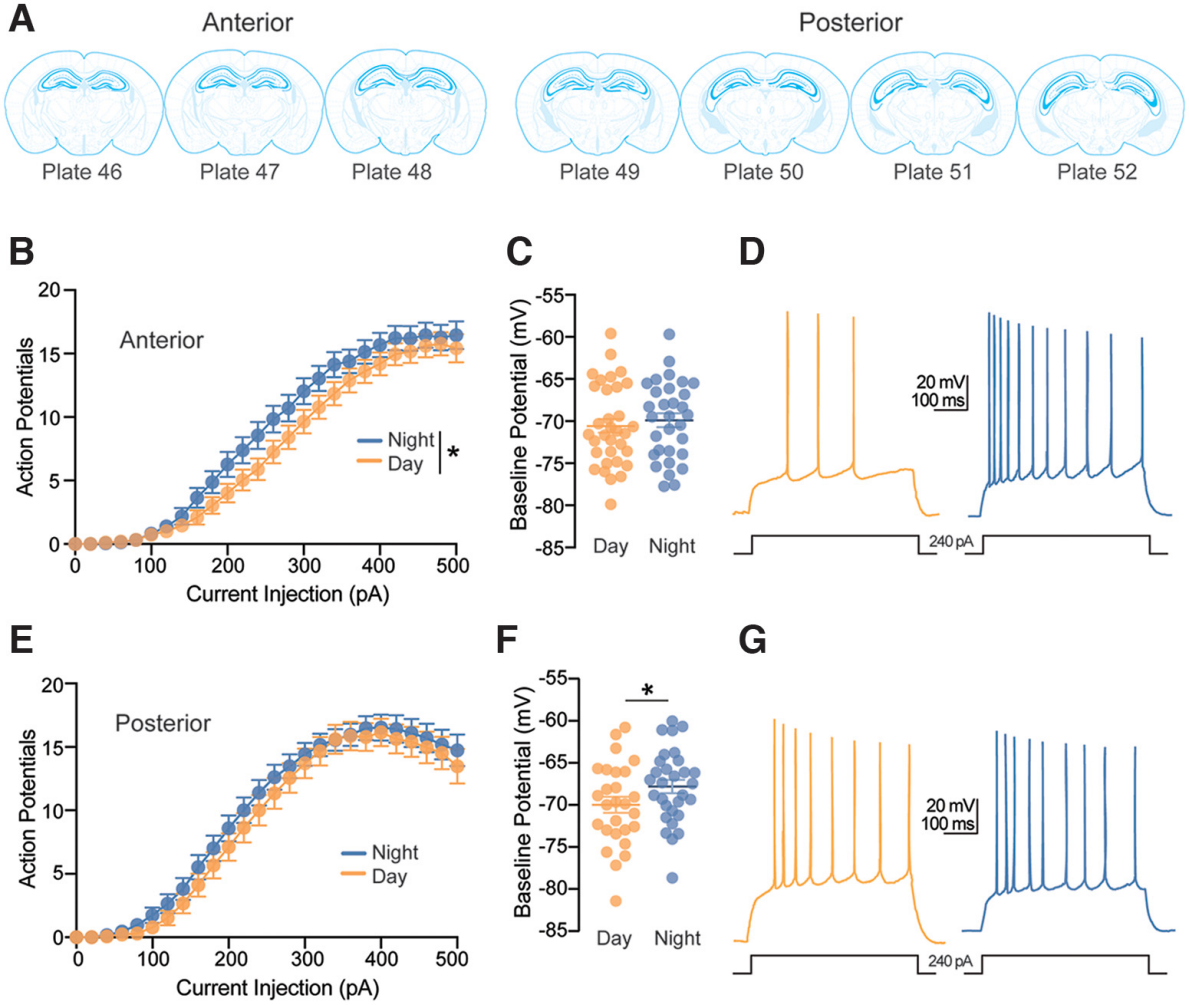
Excitability of CA1 pyramidal neurons depends on time-of-day but not sex. ***A***, Schematic locations of CA1 pyramidal neurons across the anterior-posterior axis in reference to the Allen Brain Atlas. Cells were considered “anterior” if recorded in hippocampal slices that corresponded to plate 48 or lower in the Allen brain atlas, and “posterior” if they corresponded to plate 49 or higher. ***B***, Average number of action potentials fired in response to increasing depolarizing current injections in neurons recorded from anterior slices from 14 mice at night (*n *=* *33 cells) and 11 mice during the day (*n *=* *33 cells; time-of-day: *p =* *0.046). ***C***, Scatterplot of individual values with mean ± SEM of baseline membrane potentials of neurons recorded from anterior slices (time-of-day: ns *p *=* *0.535). ***D***, Example traces of a 240-pA current step response in anterior slices from two male mice recorded during the day and night. Scale bars represent 20 mV and 100 ms. ***E***, Average number of action potentials fired in response to increasing depolarizing current injections in neurons recorded from posterior slices from 11 mice at night (*n *=* *27 cells) and 11 mice during the day (*n *=* *27 cells; time-of-day: ns *p *=* *0.484). ***F***, Scatterplot of individual values with mean ± SEM of baseline membrane potentials of neurons recorded from posterior slices (time-of-day: *p =* *0.044). ***G***, Example traces of a 240-pA current step response in posterior slices from two male mice recorded during the day and night. Scale bars represent 20 mV and 100 ms. In all plots, blue codes recordings at night and orange codes recordings during the day. SEM, standard error of the mean. All statistical tests were performed with a two-way RM-ANOVA. Data were shown as means ± SEMs. For a detailed statistical summary, see Extended Data Table 1-1.

Data were collected from neurons throughout the anterior-posterior axis of the hippocampus. Previously published studies found electrophysiological diversity in CA1 pyramidal neurons that is dependent on position across axes (Spruston, 2008; Marcelin et al., 2012; Dougherty et al., 2012, 2013; Hönigsperger et al., 2015; Kim and Johnston, 2015; Malik et al., 2016; Milior et al., 2016); thus, we chose to account for this factor by classifying all neurons as either “anterior” or “posterior” based on the coronal section anatomy (Allen Reference Atlas from https://atlas.brain-map.org/; Fig. 7*A*). When we included the anterior-posterior axis as a factor in our initial ANOVA model assessing number of action potentials per current step, we found that the largest contributing factor was region (*p *=* *0.005, main effect, four-way RM-ANOVA). Additionally, significant regional differences were found for input resistance (A: 64.27 ± 1.95 MΩ, P: 75.90 ± 2.47 MΩ, *p *<* *0.001, three-way ANOVA), rheobase (A: 146.69 ± 7.50 pA, P: 112.32 ± 6.10 pA, *p < *0.001, three-way ANOVA), and Imax, or current at which neurons fired at maximum frequency (A: 417.27 ± 9.29 pA. P: 386.90 ± 10.82 pA, *p *=* *0.047, three-way ANOVA). These differences between anterior and posterior neurons aligned with previously published studies that found diversity among dorsal and ventral CA1 pyramidal neurons. While our coronal slice preparation did not allow us to truly isolate ventral CA1, posterior sections are more likely to include some ventral CA1 pyramidal neurons. Indeed, we found that posterior neurons had properties consistent with previously published data in ventral CA1 pyramidal neurons, while anterior neurons were similar to dorsal pyramidal neurons (Dougherty et al., 2012; Malik et al., 2016).

In order to examine sex and time-of-day differences, we stratified our dataset based on region of the anterior-posterior axis followed by three-way factorial ANOVA. We found that sex had no significant effect on number of action potentials generated in response to depolarizing current injections in cells recorded from either anterior (*p *=* *0.321, main effect of sex, three-way RM-ANOVA) nor posterior slices (*p *=* *0.568, main effect of sex, three-way RM-ANOVA); thus, data from both sexes were pooled for final analysis. Anterior cells recorded at night fired more action potentials than those recoded during the day (*p *=* *0.046, main effect of time-of-day, two-way RM-ANOVA; Fig. 7*B*). However, posterior cells displayed no statistical day-night difference in the number of action potentials fired (*p *=* *0.484, main effect of time-of-day, two-way RM-ANOVA; Fig. 7*E*).

Examination of the baseline membrane potential revealed no differences in sex or time-of-day in cells recorded from anterior slices (*p *=* *0.572 and 0.535, main effects of sex and time-of-day, respectively, two-way ANOVA; Fig. 7*C*). However, membrane potentials of cells recorded from posterior slices at night were more depolarized than those recorded during the day, regardless of sex (*p *=* *0.044, main effect of time-of-day, two-way ANOVA; Fig. 7*F*). We examined 15 additional membrane properties, including action potential properties of single action potentials elicited by rheobase current injection, sag, and input resistance (Table 1). There was a significant effect of sex on action potential rise time in anterior neurons (male: 0.295 ± 0.01 ms, female: 0.275 ± 0.01 ms; *p *=* *0.047, two-way ANOVA). No other parameters reached statistical significance (Extended Data Table 1-1).

Given that action potential firing and membrane potential are both measures of neuronal excitability, we can conclude that CA1 pyramidal neurons across the hippocampal circuit are more excitable at night overall, but the mechanisms underlying this nighttime increase in excitability may vary across the hippocampal anterior-posterior axis.

### Day-night differences in CA1 pyramidal neuron excitability are not intrinsic

**Figure 8.**
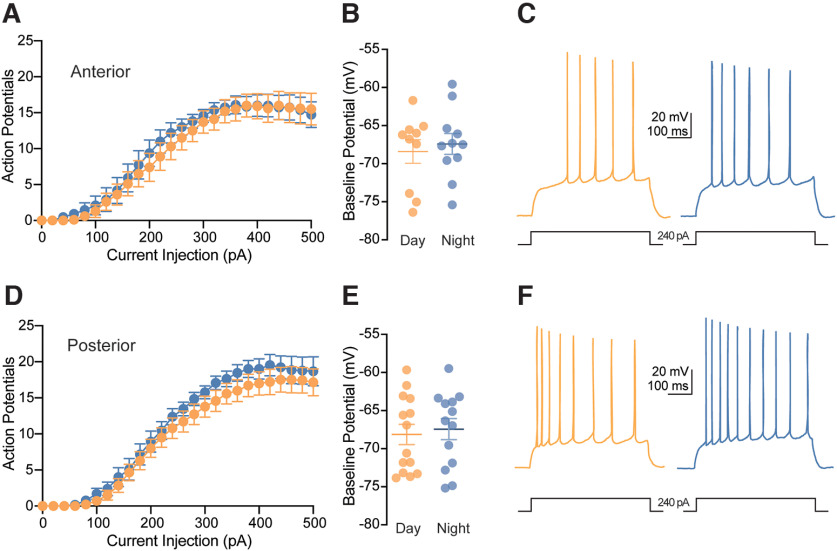
Diurnal differences in CA1 pyramidal neuron excitability are dependent on synaptic inputs. ***A***, Average number of action potentials fired in response to increasing depolarizing current injections in neurons recorded from anterior slices from six mice at night (*n *=* *11 cells) and six mice during the day (*n *=* *10 cells) in the presence of synaptic antagonists (time-of-day: ns *p *=* *0.933). ***B***, Scatterplot of individual values with mean ± SEM of baseline membrane potentials of neurons recorded from anterior slices (time-of-day: ns *p *=* *0.896). ***C***, Example traces of 240-pA current step response in anterior slices from two male mice recorded during the day and night in the presence of synaptic antagonists. Scale bars represent 20 mV and 100 ms. ***D***, Average number of action potentials fired in response to increasing depolarizing current injections in neurons recorded from posterior from six mice at night (*n *=* *13 cells) and six mice during the day (*n *=* *14 cells; time-of-day: ns *p *=* *0.569). ***E***, Scatterplot of individual values with mean ± SEM of baseline membrane potentials of neurons recorded from posterior slices (time-of-day: ns *p* = 0.999). ***F***, Example traces of a 240-pA current step response in posterior slices from two male mice recorded during the day and night in the presence of synaptic antagonists. Scale bars represent 20 mV and 100 ms. In all plots, blue codes recordings at night and orange codes recordings during the day. SEM, standard error of the mean; ns, not significant. All statistical tests were performed with a two-way RM-ANOVA. Data were shown as means ± SEMs. For a detailed statistical summary, see Extended Data Table 1-1.

As the observed day-night variation in excitability could be driven by synaptic and/or intrinsic factors, we again assessed excitability in a separate cohort of animals in the presence of synaptic antagonists (CPP, NBQX, GBZ) to isolate the CA1 pyramidal neuron from the circuit. Under these conditions, we found no significant effect of time-of-day in the number of action potentials generated in response to depolarizing current injections in cells recorded from both anterior and posterior slices (*p *=* *0.933 for anterior and *p *=* *0.569 for posterior, main effect of time-of-day, two-way RM-ANOVA; Fig. 8*A*,*D*). Furthermore, baseline potentials under these conditions, were not affected by time-of-day or sex in cells recorded from either anterior (*p *=* *0.896 and 0.888, main effect of sex and time-of-day, respectively, two-way ANOVA; Fig. 8*B*) or posterior slices (*p *=* *0.999 and 0.702, main effect of sex and time-of-day, respectively, two-way ANOVA; Fig. 8*E*). Additional membrane properties in the presence of synaptic antagonists were investigated, including action potential properties of single action potentials elicited by rheobase current injection, sag, and input resistance (Table 2). Both fast-action and medium-action potential hyperpolarization (fAHP and mAHP) were greater during the day (fAHP: −5.77 ± 0.74 mV, mAHP: −9.29 ± 0.65 mV), compared with night in anterior neurons (fAHP: −4.08 ± 0.51 mV, mAHP: −7.06 ± 0.59 mV; *p *=* *0.044 and 0.024 for fAHP and mAHP, two-way ANOVA; Table 2). In anterior neurons, fAHP was greater in males (−5.827 ± 0.56 mV) compared with females (−3.98 ± 0.58 mV; *p *=* *0.034, two-way ANOVA; Table 2). No other parameters in either anterior or posterior neurons reached statistical significance (Extended Data Table 1-1).

Overall, the absence of enhanced nighttime neuronal excitability (membrane potential and action potential firing rates) in the presence of synaptic antagonists suggests that diurnal differences fast excitatory and/or inhibitory synaptic input at least partially contribute to the nighttime enhancement of excitability.

## Discussion

Here, we examined the effects of sex and time-of-day on multiple facets of hippocampal physiology, from behavior to individual neuronal physiology. We demonstrate that time-of-day impacts spatial learning and memory, LTP magnitude, synaptic inhibition onto CA1 pyramidal neurons, and CA1 pyramidal neuronal excitability. We found that sex was most influential on day-night differences in OLM performance, while its effect on synaptic transmission, LTP magnitude, and neuronal excitability were subtle or absent completely. Additionally, we found that position along the anterior-posterior axis significantly impacts CA1 pyramidal neuron excitably. These findings illustrate the complexity of the hippocampal network and the importance of considering factors like sex and time-of-day in future studies.

While circadian rhythms regulate LTP and hippocampal-dependent learning and memory, the role of sex on diurnal differences in these processes was previously unknown. Surprisingly, we found that circadian rhythms regulate hippocampal-dependent memory performance in a sex-dependent manner. Male mice performed better on the OLM task at night, as previously reported (Takahashi et al., 2013; Snider et al., 2016). Female mice, however, performed better during the day. It is unclear why female mice would perform better during their inactive period, but a possible explanation could be the estrous cycle, which was not controlled for in the present study. While more research in naturally cycling females is needed to make definitive conclusions about the specific impact of the estrous cycle on hippocampus-dependent spatial memory, studies in rats found that females in proestrus and estrous outperformed those in diestrus on object-recognition and object-placement tasks (Frye et al., 2007; Paris and Frye, 2008). Indeed, estrogen levels do impact performance on hippocampus-dependent memory tasks and administration of exogenous estradiol enhances performance on hippocampus-dependent memory tasks (Luine et al., 2003; Li et al., 2004; Phan et al., 2012; Vedder et al., 2013, 2014; Tuscher et al., 2015). Understanding how the estrous cycle and the circadian cycle converge to modulate cognition in females will be an interesting and important topic for future research.

Interestingly, we found that these sex effects on diurnal differences in OLM performance did not extend to diurnal regulation of LTP, which is considered a cellular correlate of learning and memory. It is important to note that CT and ZT times used in the two experiments did not perfectly mirror one another. OLM assays were conducted in the middle the subjective day and night periods in constant darkness (CT four and CT 16), as adapted from (Snider et al., 2016), while electrophysiology experiments examined early day and early night in a LD cycle (ZT 1–6 and ZT 12–18). Nevertheless, this somewhat unexpected finding could be an example of how the same physiological process (LTP) can be used to achieve different outcomes depending on context (males vs females). Additionally, while the OLM task we chose is hippocampus dependent (Barker and Warburton, 2011); learning and memory are complex processes that can rely on multiple memory systems, and perhaps females rely more heavily on other circuits compared with males. Moreover, the estrous cycle can influence learning strategies and the relative contributions of different memory circuits in females (Korol et al., 2004). Therefore, it is entirely possible that the sex-dependent effects observed in the OLM task are mediated by a mechanism other than LTP at the CA3-CA1 synapse. It is also possible that the high-frequency stimulation used, as opposed to a more physiological stimulation (i.e., theta-burst), may have occluded detection of sex-dependent regulation of LTP. An additional limitation of the OLM assay worth noting is possible disruption to behavioral rhythms resulting from repeated handling (5 min/d) 4 d before training and testing. Regardless, these results exemplify the importance of accounting for both sex and time-of-day when designing research studies and interpreting results.

Previous work from our laboratory revealed that sIPSCs onto CA1 pyramidal neurons exhibit diurnal differences and that those diurnal differences are lost in a mouse model of Alzheimer’s disease (Fusilier et al., 2021). Here, we replicated and expanded on previous findings by examining sIPSCs in both sexes and conducting additional experiments in the presence of TTX (mIPSCs) to begin to identify presynaptic and postsynaptic mechanisms contributing to diurnal differences. The lack of diurnal differences in mIPSCs IEI suggest that that action potential-dependent inhibition onto CA1 pyramidal cells is greater during the day than night in both male and female mice. Given that these inhibitory currents were pharmacologically isolated with glutamate receptor antagonists, it is likely that increased daytime interneuron activity is spontaneously generated. Indeed, prior reports suggest that some interneurons in area CA1 are spontaneously active (Sik et al., 1995; Maccaferri and McBain, 1996; Oliva et al., 2000; Amilhon et al., 2015; Huh et al., 2016; Miri et al., 2018); however, definitive evidence of diurnal variation in spontaneous interneuron firing in hippocampus is lacking and will be an important area of future study that could provide insight into circadian dysfunction associated with diseases involving hyperexcitability of the hippocampal network, including Alzheimer’s disease and epilepsy.

In addition to receiving inhibitory input from local interneuron populations, major excitatory input onto CA1 pyramidal cells arrives from the axons of principal neurons of the downstream area CA3 (Schaffer collaterals; CA3 pyramidal cells) or from the entorhinal cortex (temperoammonic pathway). Examination of sEPSCs revealed a trend for excitatory input onto CA1 pyramidal cells to be greater at night than during the day. In the absence of action potential-dependent neurotransmitter release, this phenomenon persists in males, but not in females, suggesting that increased nighttime excitation in females is likely action potential driven. CA3 pyramidal cells also exhibit diurnal differences in excitability, such that night cells exhibit larger calcium current, decreased afterhyperpolarization, and reduced spike frequency adaptation compared with day cells (Kole et al., 2001). This increased nighttime CA3 pyramidal cell excitability could translate to increased sEPSC onto CA1 pyramidal cells at night compared with day.

We next wanted to examine how diurnal variation in excitatory and inhibitory synaptic transmission might impact CA1 pyramidal neuron excitability. A previous study in rats found that membrane excitability oscillated across circadian time, neurons were more depolarized during the subjective late night/subjective early day (Naseri Kouzehgarani et al., 2020). A recent study found increased nighttime excitability in mouse CA1 pyramidal neurons (Fusilier et al., 2021). Here, we replicated previous findings and expanded our study to account for potential sex differences and the influence of the anterior-posterior hippocampal axis. Overall, we found that, in an intact synaptic circuit (i.e., without synaptic antagonists), CA1 pyramidal neurons were more excitable at night compared with day, regardless of sex. Interestingly, nighttime enhancement of excitability was not uniform across the hippocampal anterior-posterior axis. While neurons recorded from anterior slices fired more action potentials in response to depolarizing current injections and displayed no diurnal difference in baseline membrane potential, posterior neurons were more depolarized at night but did not display a statistically significant nighttime increase in number of action potentials fired. These findings suggest that the underlying mechanisms for nighttime enhancement of neuronal excitability may be different depending on location across the anterior-posterior axis. While our coronal slice preparation meant we were unable to truly isolate ventral hippocampus from dorsal hippocampus, we observed that neurons from posterior slices displayed characteristics consistent with previously published data collected from ventral CA1 pyramidal neurons, while neurons from anterior slices were consistent with data examining dorsal CA1 pyramidal neurons (Malik et al., 2016; Milior et al., 2016). Specifically, posterior (ventral-like) neurons were more excitable compared with anterior neurons, reaching max firing rate earlier than anterior neurons, had higher input resistance, and lower rheobase values compared with anterior (dorsal-like) neurons. Given known differences in dendritic morphology and ion channel expression in dorsal versus ventral hippocampal neurons (Bannerman et al., 2004; Fanselow and Dong, 2010; Marcelin et al., 2012; Dougherty et al., 2012, 2013; Hönigsperger et al., 2015; Kim and Johnston, 2015; Malik et al., 2016; Milior et al., 2016; Soltesz and Losonczy, 2018), it is unsurprising that mechanisms underlying diurnal differences in excitability may be different across these populations. The absence of day-night differences of neuronal excitability in the presence of synaptic antagonists suggests that synaptic inputs at least partially contribute to the nighttime enhancement of excitability. However, future experiments are needed to determine the specific role of both synaptic and intrinsic factors in regulating diurnal differences of physiology in CA1 pyramidal neurons. Additionally, the difference between anterior and posterior cells was lessened in the presence of synaptic antagonists, suggesting synaptic factors could be at least partially responsible for some of the regional differences we observed. It will be interesting to narrow down how the expression and function of various neurotransmitter receptors and ion channels are modulated across both time-of-day and location along the longitudinal axis. A benefit of the blind patch technique used in the present study is the ability to collect data from neurons located deep below the surface of the tissue, where cell health and viability is greatest but visualized targeting of neurons would be difficult. However, this approach prohibits targeting specific neuronal subpopulations; thus, pyramidal neurons throughout all areas of the CA1 pyramidal cell layer were included in the study. Future studies examining time-of-day and sex effects across different subpopulations of CA1 pyramidal cells (e.g., deep vs superficial neurons) will be informative for understanding cell-type-specific physiology.

The hippocampus is one of the most studied and well-characterized circuits in the mammalian brain. However, most of the knowledge about how this circuit functions is based on studies conducted during the day in, mostly male, nocturnal rodents. Failing to account for factors like time-of-day and sex, leads to an incomplete picture hippocampal physiology and how it dynamically functions across multiple contexts. Sex and time-of-day are especially important considerations for the translational relevancy of studying the hippocampus in models of diseases like Alzheimer’s or epilepsy, which are influenced by circadian rhythms and can affect men and women differently. It is important to note that, except for experiments testing OLM, all experiments in this study were conducted in a light-dark cycle. Therefore, future studies in constant conditions are needed to determine the role of the circadian system on observed diurnal differences. While only two timepoints were examined here, it will be interesting and helpful to determine how hippocampal physiology is dynamic across multiple time points in the circadian cycle in future studies. In conclusion, this study reveals diurnal variation in hippocampal synaptic and neuronal function, and underscores the importance of considering sex, circadian rhythms, and neuronal heterogeneity within a brain region in the study of neural circuits.

## Synthesis

Reviewing Editor: Michaël Zugaro, CNRS, Collège de France, Inserm Decisions are customarily a result of the Reviewing Editor and the peer reviewers coming together and discussing their recommendations until a consensus is reached. When revisions are invited, a fact-based synthesis statement explaining their decision and outlining what is needed to prepare a revision will be listed below. The following reviewer(s) agreed to reveal their identity: NONE.

It is known that hippocampal neurons express clock genes, can vary in the electrophysiological properties over 24h, and that cognitive performance can vary over the circadian cycle. This study examines the impact of gender and time of day on multiple aspects of anatomically distinct subregions of the hippocampus, from behavior to individual neuronal physiology.

The authors aimed to determine if the the circadian cycle (time of day) has an effect on excitability of CA1 pyramidal neurons, spatial learning and memory using an object-linked memory task, LTP magnitude, synaptic inhibition on CA1 pyramidal neurons. Sex had the greatest impact on changes in learning and memory between day and night, with better OLM performance in males at night, but during the day in female mice.

On the other hand, its impact on synaptic transmission and LTP magnitude was weaker. LTP at CA3-CA1 synapses was greater at night in both male and female mice. Patch recordings of CA1 neurons showed that the polarity of synaptic inputs to these neurons differed, with the frequency and magnitude of inhibitory inputs being larger during the day than the night in both male and female mice. In contrast, the frequency and amplitude of excitatory input was larger in female mice than male mice at both day and night time points. In both sexes of mice, the excitability of CA1 pyramidal neurons was greater at night and this was associated with the relative balance of excitatory to inhibitory inputs at this time. Furthermore, the position along the anterior-posterior axis has a considerable impact on the excitability of CA1 pyramidal neurons. The authors concluded that diurnal variation in hippocampal synaptic and neuronal function is important to consider when studying neural circuits, as well as sex, circadian rhythms, and neuronal heterogeneity within a brain region.

This study is well-designed, solid and interesting, however serveral questions must be addressed to provide strong evidence for the claims.

There are some points to consider:

1. It is not clear from the description how mice in constant dark (under dim red illumination) use visual cues when seeking out the object. The researchers need to provide more details here. How visual is this task? Can the mice detect the stripes and shape on the walls when in dim red? How were the mice transported to the test arenas? How does this performance compare to mice tested in normal lighting conditions?

2. Related to 1), mice ordinarily rest more during the day/subjective night. With repeated testing, the mice tested during the day may be incurring sleep debt as they will not be able to sleep as much as mice tested during the night. The researchers should comment on the implications of this as sleep deprivation will lead to alterations in hippocampal excitability and function. Perhaps what the researchers have found is differences in how sleep deprived the two sexes of mice are?

3. Did the researchers assess if and how entrainment to LD of mice may be altered by this cognitive testing regimen?

4. Are the mice group-housed during the study and if so, how many mice per cage?

5. While there was no effect of time of day or gender on overall exploration time, this does not necessarily mean that their locomotor activity was comparable. Mice are nocturnal animals, and their locomotor activity varies greatly from day to night. In addition, sex-related changes in locomotor activity in a novel environment have been identified. Is it feasible that the results are related to their locomotor activity, given that there is no association between LTP and memory task in female mice? During the task, the authors need to demonstrate the link between locomotor activity and memory performance.

6. During the habituation period (Days 3-5), mice experienced some form of sleep disturbance or sleep deprivation. Will this have an impact on their ability to complete the task? Please also provide the total exploration time (sec) for male vs. female and day vs. night in Figure 1 as well.

7. Mice were subjected to three days of habituation in constant darkness (DD). The authors then completed the OLM task. All electrophysiological measurements, however, were acquired at ZT 0-1 (day) or ZT 11-12 (night). One potential issue is that the results from the electrophysiological experiments did not match the data from the behavior experiments. Sleep disruption is a significant stressor that can affect everything from behavioral to synaptic and neuronal excitability. The authors should provide data demonstrating similar outcomes in the absence of DD habituation: LTP and neuronal excitability (e.g., the number of action potential at rest) for male vs. female and day vs. night.

8. Where do the authors measure the slope of fEPSP? Please clarify in the method section.

9. The authors used a bar graph from Figure 3 to Figure 6. Please replace the bar graph with a scatter plot. It may provide extra information to interpret the results of electrophysiological data.

10. Please double-check the statistical analysis in Figures 3B and 3C (male) and Figures 4B and 4C (male). Figures 3B and 3C (male) appear to have no significant impacts, but Figures 4B and 4C appear to have a considerable effect (male).

11. The magnitudes of neuronal excitability (e.g., APs and input resistance) are influenced by neuronal morphology and their longitudinal location within the CA1 area. Figures 7 and 8 demonstrate action potential firing at rest and resting membrane potential in two dorsal hippocampus regions (anterior and posterior dorsal hippocampi). The aAuthors concluded that increased nighttime neuronal excitability of CA1 neurons is driven primarily by synaptic, not intrinsic, factors. Before drawing this conclusion, the authors should provide more details, such as the location of recorded neurons within the CA1 region, action potential properties (e.g., threshold at rest), and input resistance at rest and at holding membrane potential (e.g., at -65 or -70 mV) with and without synaptic blockers.

12. In fEPSP and EPSC/IPSC experiments, please clarify the position of recorded region/neurons of CA1 area.

13. Please rewrite the sentence at line 388-389, e.g. “we divided cells into anterior and posterior dorsal hippocampal groups based on coronal section anatomy”.

14. In line 520-523, please clarify which aspects of the study are consistent with previously published evidence from ventral CA1 neurons: input resistance, resting membrane potential, and threshold?
