# Supplementary figures and images for: Examination of Diurnal Variation and Sex Differences in Hippocampal Neurophysiology and Spatial Memory
Source: eNeuro. 2022 Nov 8;9(6):ENEURO.0124-22.2022. doi: 10.1523/ENEURO.0124-22.2022 (PMC9668349; doi:10.1523/ENEURO.0124-22.2022)

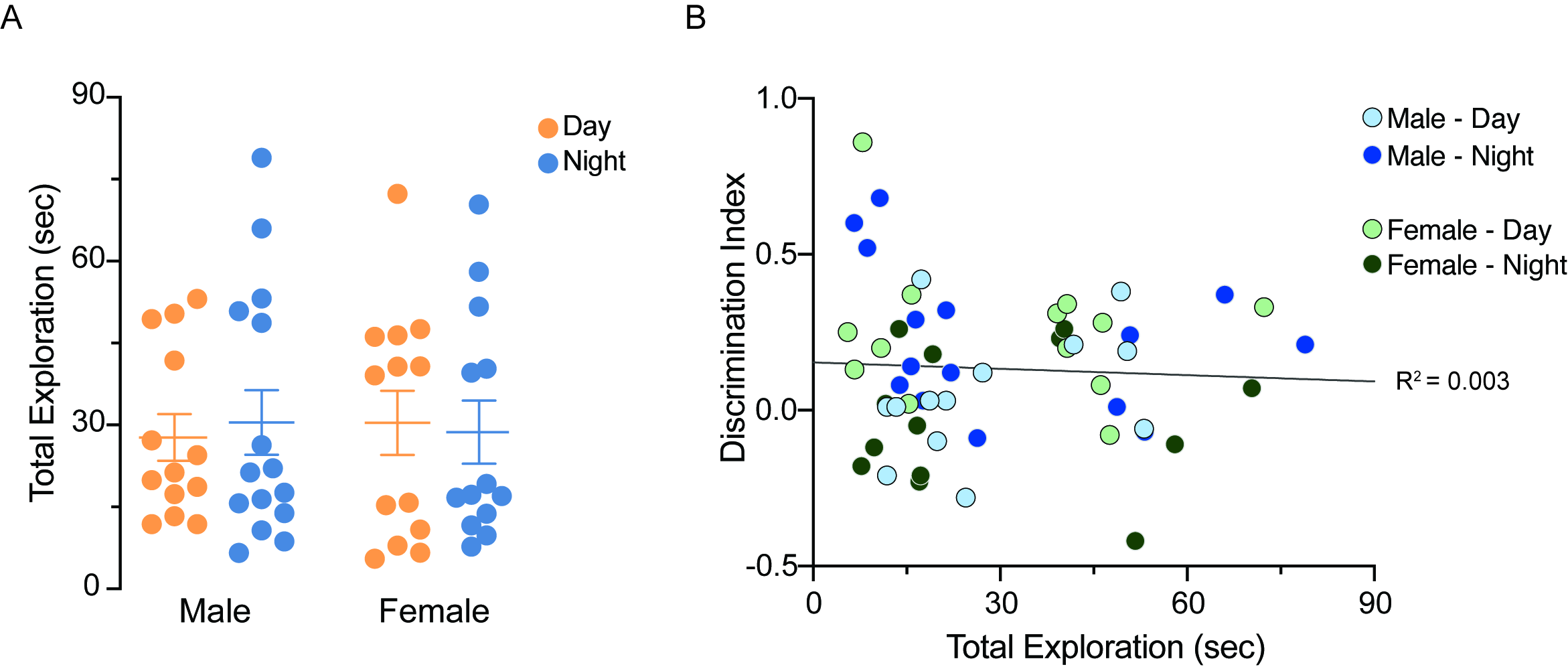

Supplement: Extended Data Figure 1-1 — Total exploration is not different across groups and does not predict OLM performance. A, Total exploration time for individual mice during the testing phase with mean ± SEM across day and night in both sexes (time-of-day: ns p = 0.926, sex: ns p = 0.936, interaction: ns p = 0.692, two-way ANOVA). There was no difference in distribution of high explorers (total exploration time > 35 s) and low explorers (total exploration time < 35 s) across four groups (p = 0.619, Pearson’s χ2). B, Correlation between total exploration times and DI scores during test phase (ns p = 0.704, r = –0.053, Pearson’s correlation). Male night (dark blue) n = 13 mice; male day (light blue), n = 13 mice; female day (light green), n = 15 mice; female night (dark green), n = 13 mice. For a detailed statistical summary, see Extended Data Table 1-1. Download Figure 1-1, TIF file [file enu-eN-NWR-0124-22-s03.tif]
